# Who and why do researchers opt to publish in post-publication peer review platforms? - findings from a review and survey of F1000 Research

**DOI:** 10.12688/f1000research.15436.1

**Published:** 2018-06-27

**Authors:** Jamie Kirkham, David Moher

**Affiliations:** 1 Department of Biostatistics, University of Liverpool, Liverpool, L69 3GL, UK; 2 Centre for Journalology, Ottawa Hospital Research Institute, Ottawa, Canada

**Keywords:** F1000 Research, Journalology, Peer Review, Rapid Publication

## Abstract

**Background:  **Preprint servers and alternative publication platforms enable authors to accelerate the dissemination of their research.  In recent years there has been an exponential increase in the use of such servers and platforms in the biomedical sciences, although little is known about who, why and what experiences researchers have with publishing on such platforms.  In this article we explore one of these alternative publication platforms, *F1000 Research, *which offers immediate publication followed by post-publication peer review.

**Methods: **From an unselected cohort of articles published between 13 ^th^ July 2012 and 30 ^th^ November 2017 in *F1000 Research*, we provided a summary of who and what was published on this platform and calculated the percentage of published articles that had been indexed on a bibliographic database ( *PubMed*) following successful post-publication peer review.  We also surveyed corresponding authors to further understand the rationale and experiences of those that have published using this platform.

**Results: **A total of 1865 articles had been published in the study cohort period, of which 80% (n=1488) had successfully undergone peer review and were indexed on *PubMed *within a minimum period of six months since first publication. Nearly three-quarters of articles passed the peer review process with their initial submission.  Survey responses were received from 296 corresponding authors. Open access, open peer review and the speed of publication were the three main reasons why authors opted to publish with *F1000 Research*.

**Conclusions: **Many who published with *F1000 Research *had a positive experience and indicated that they would publish again with this same platform in the future.  Nevertheless, there remained some concerns about the peer review process and the quality of the articles that were published.

## Introduction

The conventional method of journal publication involves manuscript submission, peer review and editorial oversight, revision and publication. While this process is presumed to ensure the scientific integrity of the research undertaken, the availability of the research findings entering the public domain may take several months or even years depending on factors such as a journal editors decision to publish or reject an article, peer reviewer availability or a journal’s publication frequency. The efficiency of peer review was underlined in a recent peer review survey conducted in 2018 by ASAPbio ( Accelerating Science and Publication in biology). The survey revealed that for their most recent published article, about 50% of the authors surveyed (132/259) submitted their article to two or more journals, with 7% (18/259) submitting to five or more [ http://asapbio.org/peer-review/survey]. This process can result in a substantial delay in research findings entering the public domain. Traditionally, authors have not been able to add such research findings to their curriculum vitae and/or grant applications. In some scientific fields such as pandemics or humanitarian emergencies, the time to deliver research findings may be as equally as important as research quality, and may be critical to health care provision.

While some journals may offer a ‘fast track’ service to publication, preprint servers offer rapid publication on all articles but without systematic refereeing, which brings significant benefits to the authors, the presentation of the article and the readers. The profile of preprint servers is increasing, with many major funders (e.g. Wellcome Trust and National Institute of Health (NIH)), now endorsing the use of preprint servers, particularly for grant applications [ https://wellcome.ac.uk/news/we-now-accept-preprints-grant-applications, http://www.sciencemag.org/news/2017/03/nih-enables-investigators-include-draft-preprints-grant-proposals]. 

However, due to lack of peer review, preprint servers in a life sciences setting have been criticised as they may lack quality and subsequently have the potential to report flawed research which may harm patients ^1^. To improve scientific integrity, new emerging options towards publication are being considered under the Open Science Initiative. An example includes peer review before the results are known ‘registered reports’, which aims to eliminate questionable research practices and poor research design [ https://cos.io/rr/]. The registered reported publishing format is currently being used by over 100 journals.

*F1000 Research* is an example of an alternative ‘platform’ which offers the advantages of a preprint server in terms of immediate publishing on a variety of research article types linked to biomedical research, with the added advantage of post-publication open peer review. Once peer review is complete (at least two approved referee reviews, or one approved plus two approved with reservations reviews) the article is subsequently indexed in a bibliographic database such as *PubMed*. 

As of May 2018, *F1000 Research* has published over 2000 articles since its inception in 2012. Little is known about who, and the reasons why authors publish in *F1000 Research*. The aim of this study is to provide a descriptive summary of the research that has been published in *F1000 Research,* and to determine how much of this published research has been accepted for bibliographic database indexing. We also survey authors who have published in *F1000 Research* to further understand the rationale and experiences of those that have published using this publication platform. 

## Methods

We studied a cohort of all article types that were first published on *F1000 Research* between 13 ^th^ July 2012 (earliest publication) and 30 ^th^ November 2017. A data extraction form was developed and piloted on the first page of 20 listed publications. For each article, the following information was extracted; article type, the year of publication, funding sources, the country of the first listed corresponding author and the peer review status. The peer review status for all articles was last verified on May 30 ^th^ 2018, i.e. six months after the last published article in the study cohort. At the same time, we also checked whether articles were indexed on the bibliographic database, *PubMed*.

Articles published in *F1000 Research* between the period 13 ^th^ July 2012 and 30 ^th^ November 2017 Click here for additional data file. Copyright: © 2018 Kirkham J and Moher D 2018 Data associated with the article are available under the terms of the Creative Commons Zero "No rights reserved" data waiver (CC0 1.0 Public domain dedication).

### Survey of corresponding authors who have published with F1000 Research.

With the exception of Editorials and F1000 Faculty Critiques which are published by invitation only and not subject to external peer review, the first listed corresponding author of all published studies in the study cohort were contacted via a personalised email. We removed any duplicate email addresses such that a corresponding author who had published multiple articles were contacted only once. Participants were asked to participate in a short online survey with regards to their main reasons and experiences of publishing with *F1000 Research*. The survey was constructed using the online survey software, REDcap [ https://www.project-redcap.org/], and was open for responses between 6 ^th^ April 2018 and 10 ^th^ May 2018. The survey questions are available in Supplementary File 1 and reflect the importance of a series of factors that may influence the decision to submit to *F1000 Research* as rated on a five-point Likert scale ranging from ‘very important’ to ‘not important’. Similarly, on a 5-point Likert scale we asked about the importance of articles being indexed on a bibliographic database, the importance of a transparent peer review process and the likelihood that they would submit future manuscripts to *F1000 Research* or recommend the platform to others. Participants could also provide free text comments on positive or negative experiences associated with submitting or publishing with the platform. Non-responders were contacted periodically if a response to the survey was not received. The data were presented as the frequency distribution for each level of response. All positive and negative experiences were independently reviewed by both authors and categorized into common topics. Any discrepancies were solved via discussion. 

The University of Liverpool Ethics Committee was consulted and granted ethical approval for this study (Reference 3233). Informed consent was assumed if a participant responded to the survey.

## Results

### Summary of articles publishing in F1000 Research

**Figure 1.**
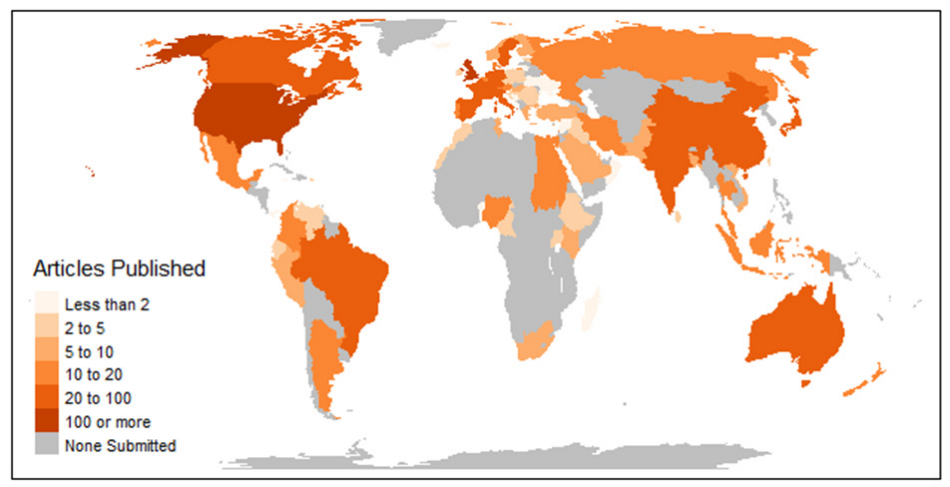
Map of corresponding author locations of all articles published in F1000 Research (13 ^th^ July 2012 to 30 ^th^ November 2017).

A total of 1865 articles were published in *F1000 Research* between the period 13 ^th^ July 2012 and 30 ^th^ November 2017. Just over a third of articles published were research articles (677/1865; 36%) with no more than 10% of the remaining articles published representing a different article type ( Table 1). The majority of articles published received non-commercial funding (1054/1865; 57%), while a large proportion also declared no funding source (745/1865; 40%). The first corresponding author in nearly 80% of articles published were from high income countries (1480/1865; 79%) and less than 2% were from low income countries ( Table 1, Figure 1). The six countries with more than 50 articles published were USA (618 articles), UK (232 articles), Germany (91 articles), Australia (84 articles), India (82 articles) and Canada (78 articles). There appeared to be a gradual increase in the number of articles published over time with over 400 articles published in each of the last two years in the study cohort ( Table 1).

**Table 1.** Article characteristics of all articles published in F1000 Research (13 July 2012 to 30 ^th^ November 2017).

Article Characteristics	N=1865 (%)	Article Characteristics	N=1865 (%)
**Article Type:**		**Funding ^a^:**	
Antibody Validation Article	13 (0.7)	Commercial	66 (3.5)
Case Report	192 (10.3)	Non-Commercial	1054 (56.5)
Clinical Practice Article	18 (1.0)	None	745 (39.9)
Commentary	23 (1.2)	**Corresponding Author Location ^b^:**	
Correspondence	33 (1.8)	Low Income Country	30 (1.6)
Data Article	8 (0.4)	Lower Middle Income Country	181 (9.7)
Data Note	34 (1.8)	Upper Middle Income Country	174 (9.3)
Editorial	51 (2.7)	High Income Country	1480 (79.4)
F1000 Faculty Critique	7 (0.4)	**Year submitted:**	
Method Article	104 (5.6)	2012 (earliest submission 13 ^th^ July)	72 (3.9)
Observation Article	15 (0.8)	2013	287 (15.4)
Opinion Article	174 (9.3)	2014	322 (17.3)
Research Article	677 (36.3)	2015	357 (19.1)
Research Note	161 (8.6)	2016	418 (22.4)
Review	96 (5.2)	2017 (up to 30 ^th^ November)	409 (21.9)
Short Research Article	41 (2.2)		
Software Tool Article	153 (8.2)		
Study Protocol	23 (1.2)		
Systematic Review	17 (0.9)		
Web Tool	25 (1.3)		

^a^Studies that were partially funded by industry (e.g. pharmaceutical) were classified as ‘commercial funding’ ^b^Economic status was classified according to the World Bank list of economies (June 2017)

### Peer review and bibliographic database indexing

Allowing for a minimum of six months from the first publication of all articles, 80% (1488/1865) had successfully undergone peer review (with the exception of 51 editorials that were not subject to peer review) and were indexed in the bibliographic database, *PubMed* ( Table 2). For the remaining 20% of articles, the lack of indexing was because peer review was incomplete (n=317), peer review had discontinued (n=36) or the article had been removed by authors (n=3). When peer review was incomplete, the peer review process had been ongoing for over 12 months for 80% of articles (253/317). In a small number of articles (n=14) the peer review process was complete but the article had not yet appeared in *PubMed* ( Table 2). ** Of the articles that were published in *F1000 Research,* 74% (1065/1448) passed the peer review process with the initial submission, 29% (n=336) after one revision, 3% (n=42) after two revisions, while five articles required four or five revisions.

**Table 2.** Article peer review and bibliographic database indexing status of all articles published in F1000 Research (13 July 2012 to 30 ^th^ November 2017).

Submitted Article Status	N=1865 (%)
**Indexed in Bibliographic Database**	1488 (79.8)
Article underwent full external peer review	1437 ^a, b^
Editorials not subject to external peer review	51
**Not Indexed in Bibliographic Database**	377 (20.2)
Peer review incomplete	356
Peer review ongoing	*317* ^c^
Peer review discontinued	*36*
Article removed by authors	*3*
Peer review complete	14 ^d^
F1000 Faculty Critique (not indexed)	7

^a^Two of these articles were not published by F1000 Research (South Asian Journal of Cancer (case report), La Tunisie Medicale (research article)). ^b^One article was indexed on a bibliographic database but the peer review process was incomplete. ^c^253: peer review ongoing for over 12 months since the article was first submitted. ^d^Four: peer review completed but article not indexed on a bibliographic database within 12 months of last publication date.

### Survey of corresponding authors publishing with F1000 Research

After excluding 58 articles that did not undergo peer review (editorials and or F1000 Faculty Critiques), there were 1476 unique first listed corresponding author email addresses (out of 1807 articles) that were targeted in the survey. Notably two authors were listed as the same corresponding author on 18 (Germany) and 16 articles, respectively (USA/India).

Responses to the survey were received from 296 corresponding authors. An exact response rate was difficult to estimate but we approximate this to be between 25–30% given the number of returned survey emails that had invalid and/or expired email addresses or ‘out of offices’ during the period the survey was live. The majority of responders were academic affiliated (74%; 219/296), while a minor proportion represented non-profit organisations (9%; 22/296), industry (5%; 14/296) and government (7%; 22/296). The remaining 14 represented other entities such as independent self-employed private researchers, consulting agencies, schools and hospitals. There was an increasing trend in terms of the respondents research experience with 7% (21), 18% (52), 33% (98) and 42% (125) out of the 296 respondents representing trainee, early-, mid- and senior researchers, respectively.

**Figure 2.**
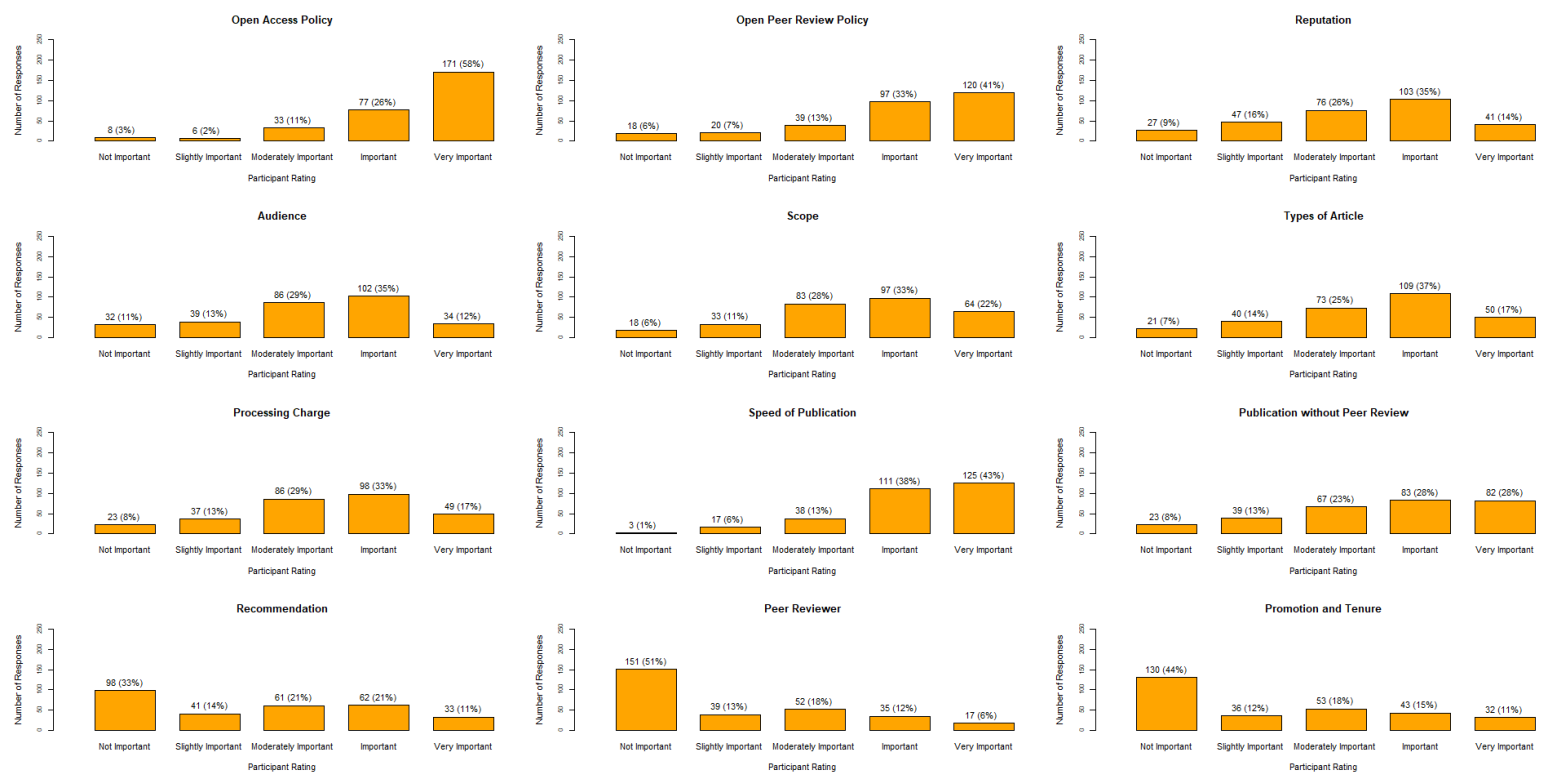
The level of importance of factors that were influential to authors when deciding to publish with *F1000 Research*.

The importance of factors that influenced an author’s decision to submit an article to *F1000 Research* are presented in Figure 2. The open access policy, open peer review policy and the speed of publication were the three top reasons for publishing with *F1000 Research*, with more than 70% of participants reporting these factors as either important or very important. Linked to peer review policy, the transparency of the *F1000 Research* peer review system that includes reviewer’s names was rated as important or very important by nearly 70% of respondents (202/295). Of less importance was the recommendation to publish in *F1000 Research* by colleagues, had previously peer reviewed for *F1000 Research* and for promotion and tenure. Nevertheless, 80% (237/295) of respondents said they would either likely or very likely recommend *F1000 Research* to a colleague. 

The respondents listed 58 additional new items relating to three themes that influenced their decision to publish with *F1000 Research*. One theme was linked to promotional activities connected directly with *F1000 Research* which included fee waivers (n=14), personal invitations to submit an article (n=2) and commissioned calls for specific articles (n=3). In another theme, the reasons were unconnected to *F1000 Research* but were a result of failures to publish in alternative journals (n=16), reasons cited included; other journals not interested in the type of article/analysis, bad reviews, biased peer review and editorial biases. In a third theme, authors chose to submit to *F1000 Research* due to specific characteristics of the publishing platform. Characteristics included, accessibility of previous versions of the article and the ability to access and respond to reviewer comments (n=5), ability to be able to publish negative findings and material on controversial topics (n=3), no size limit on articles (n=2), ability to share public datasets (n=1), quality of publication of images (n=1) and good altmetrics (n=1). The remaining ten items related to the fact that authors were intrigued to test out a new publication platform.

Nearly 90% of respondents (261/295) stated that it was either important or very important that following immediate publication with *F1000 Research*, their article was later indexed on a bibliographic database after the article was approved following peer review. The 261 corresponding authors who thought this was important listed 198 reasons for this. The most common reason was related to making the article visible and easily accessible (n=81), and ensuring that the article was sufficiently exposed (n=27) to its intended readership. Others stated that indexing enhanced the credibility and quality (n=32) of articles as it was the benchmark that the article had undergone peer review. Article indexing for assessment purposes (n=37) was also seen to be important in terms of the assessment of research impact, assessing scientists (e.g. for promotion), assessing institutes (e.g. the Research Excellence Framework in the UK) and when applying for grant income. Finally, some respondents thought that the indexing of articles was important for personal distinctions (n=21), examples included personal recognition amongst peers, citation counts and the enhancement of personal portfolios. 

### Experiences of those submitting articles to F1000 Research

The main criticism of those submitting to *F1000 Research* was related to the peer review process following publication. Authors found the process of nominating and reviewers agreeing to review challenging (n=9) given the strict criteria for selection [ https://f1000research.com/for-authors/tips-for-finding-referees], this it was felt led to typically a longer period of peer review (n=12) than most other journals. Some authors also questioned the quality of the reviews (n=9), with the suggestion that author selected reviewers may be biased or reserved in terms of lack of criticism given the public record and naming of reviewers when a platform operates an open peer review policy. A few authors felt ‘trapped’ in the peer review process and felt that once the article was published there was ‘no way out’ if reviewers could not be found or reviewers had stopped providing reviews. Publication fees was seen as major barrier for this publication platform, particularly in some areas of research where funds available for publishing is limited (n=10). The impact of articles published with *F1000 Research* was also seen as a limitation (n=16), and while this was not necessarily the authors personal concern, the perceived reputation that this would be considered a low-quality publication and poorly cited on a platform with no reputable impact factor were foreseen as issues with peers and within scientific organisations. Three authors provided condemning reviews of the platform and suggested that it ‘provided an easy opportunity to publicly criticise the work of others in an act that constitutes unwarranted bullying’ and were subsequently forced into using the platform to correct and refute the criticisms to protect personal reputation. A number of authors (n=5) also commented that the platform was difficult for editing and writing purposes and was particularly tedious when making a data deposition (n=2).

Despite some negative feedback regarding *F1000 Research*, there were also many positive responses with a number stating that this was ‘their best experience in publishing’ with the hope that this publication style becomes ‘dominant in the future’. Based on their experience, 74% (218/296) of the respondents said that they would likely or very likely submit to *F1000 Research* in the future. The speed and efficiency of publication (n=11) was the main reason that authors felt the experience was positive, while others (n=8) thought the extent and the transparency of reviews was both helpful and important. Some authors also found the editorial staff to be cooperative and professional (n=7) while other benefits included the ease of use of the platform and the standard of the publication. 

## Discussion

The explosion in the number or preprint platforms and the number of researchers submitting to preprint servers and alternative platforms in the biomedical sciences is rising exponentially [ http://www.prepubmed.org/monthly_stats/], in yet relatively little is known about them. *F1000 Research* offers a unique publishing platform, which like preprint servers offer immediate publishing but with the added advantage of post-publication peer review and eventual article indexing on a bibliometric database. The speed of the publication, alongside the open access and open peer review policy were particularly attractive traits to authors who submitted their research to this platform. Having an article indexed on a bibliometric database was seen to be important by the majority of the respondents and this study revealed that 80% of the articles achieved indexing within six months of submission to *F1000 Research*. Visibility and accessibility of research articles were deemed to be the most common reason for submission. The visibility of publishing research without peer review plays another important role. The peer review for about 15% of the articles had either been ongoing for more than a year or discontinued completely meaning that many of these articles may have contributed to the vast quantity of inaccessible unpublished literature (and the potential for publication bias) had these articles been subjected to the standard peer review before publication model. 

The *F1000 Research* publication model was not without criticisms. Some found that the peer review process took longer than standard journals because there was more emphasis on the authors rather than editors to find peer reviewers. There was also a sense that there was the potential for an article to become caught up in the process, immediate publication meant that there was limited scope to remove or submit elsewhere if peer reviewers could not be found or existing reviewers failed to provide subsequent reviews. While these criticisms may have reflected the experiences of some survey respondents, this process is not dissimilar from the many journals/publishers of standard journals which request names of peer reviewers, and in some instances release articles if peer reviewers cannot be found in a reasonable timeframe.

The majority of respondents generally saw open peer review as a good thing, but some respondents felt this process could lead to inferior and reserved poorer quality reviews that lacked criticism, this was perhaps evidenced by the fact that 75% of articles passed the peer review stage based on the first submitted version. Despite this finding, a randomised trial has found that asking a reviewer to consent to be identified to the author had no important effect on the quality of the review but it may significantly increase the likelihood of reviewers declining to review ^2^.

The strength of this study is that we evaluated a large, unselected cohort of articles that were published with *F1000 Research*. The response to the survey was quite poor with only 296 corresponding authors engaging with the survey from the potential 1476 unique email addresses identified. Nevertheless, calculating an exact response rate was particularly challenging given several hundred of those contacts were found to be invalid or expired, a consequence of targeting some authors that published their articles several years ago. Even with the potential for such response bias, the open text comments received appeared to be relatively balanced in terms positive and negative experiences of publishing with *F1000 Research* with key themes identified. There was also a general sense that the *F1000 Research* platform appeared to be ‘modern’ and yet was potentially ‘less attractive to the early career researcher’ because the need to publish in recognised journals with high impact factors is still considered the standard by the vast majority of researchers and institutes to gain promotion and tenure.

It was clear that researchers from all around the world have published on the *F1000 Research* platform. The importance of alternative publications platforms is beginning to extend beyond an authors choice to submit to them. For example, recently Public Library of Science (PLOS) have partnered with the preprint server bioRxiv, and from May 1 ^st^ 2018, authors will have the option to post their submitted manuscript on to the preprint server in order to disseminate their work prior to peer review [ http://blogs.plos.org/plos/2018/04/one-small-step-for-preprints-one-giant-step-forward-for-open-scientific-communications/]. To a large extent this mimics the idea of the already existent *F1000 Research* publication model. 

In conclusion, there is undoubtedly and increase in the use of researchers publishing their research on alternative platforms for biomedical sciences, but there still remains a level of dogma surrounding their use by many, and there remains concerns about the quality of the articles published on these platforms.

## Data availability

The data referenced by this article are under copyright with the following copyright statement: Copyright: © 2018 Kirkham J and Moher D

Data associated with the article are available under the terms of the Creative Commons Zero "No rights reserved" data waiver (CC0 1.0 Public domain dedication). 

**Dataset 1:** Articles published in *F1000 Research* between the period 13 ^th^ July 2012 and 30 ^th^ November 2017 10.5256/f1000research.15436.d208308 ^3^

## Supplementary material

Supplementary Appendix A: Survey questions sent to corresponding authors of articles published in *F1000 Research* between 13 ^th^ July 2012 and 30 ^th^ November 2017
