# Supplementary material for: Who and why do researchers opt to publish in post-publication peer review platforms? - findings from a review and survey of F1000 Research
Source: F1000Res. 2018 Jun 27;7:920. [Version 1] doi: 10.12688/f1000research.15436.1 (PMC6053701; doi:10.12688/f1000research.15436.1)
Supplement: Supplementary file 2 [file f1000research-7-16821-s0001.tgz › 1bd8f3c5-4ba8-40a5-aab7-d66cd5224c31.docx]

**SUPPLEMENTARY MATERIAL**

Supplementary Appendix A: Survey questions sent to corresponding authors of articles published in *F1000 Research* between 13^th^ July 2012 and 30^th^ November 2017

**1) Please tell us the main type of organisation that you work in**

Academic (e.g. a University)

Government

Industry (e.g. a pharmaceutical company)

Non-Profit (e.g. a charitable trust)

Other, please specify:

**2) Please indicate your stage of career**

Trainee (working under the supervision of an independent researcher)

Early career investigator (0-5 year’s independent research experience)

Mid-career investigator (5-15 year’s independent research experience)

Senior investigator (Over 15 year’s independent research experience)

**3) Please rate the importance of each factor in your decision to submit to F1000 Research [5-point scale for each, Very important, Important, Moderately Important, Slightly important, Not important]**

F1000 Research offers an open access policy

F1000 operates an open peer review policy

Reputation of F1000 Research

The audience of F1000 Research

The scope of F1000 Research

*(covers all forms of scientific content related to the* [*life sciences and medicine*](https://f1000research.com/browse)*)*

The types of article that F1000 Research publishes

*(research articles, reviews, systematic reviews, opinion articles, and correspondence, as well as data notes, case reports, clinical practice papers, study protocols, method articles, research notes, observation articles, antibody validation articles, and software tool articles)*

The processing charge cost for a F1000 Research article

*(short article US $150 [<1000 words], medium article US $500 [1000-2500], long article US $1000 [over 2500 words])*

The speed of the publication process (e.g. no delays in peer review or editorial decisions)

F1000 Research articles are professionally published without peer review (provided the article passes the initial checks)

*(initial checks include whether the article is within scope, adheres to ethical and editorial policies, is written to a standard that the article can be sent for peer review and the content is fully accessible)*

Recommendation of a colleague/mentor/supervisor

I have peer reviewed for F1000 previously

I can use pre-prints as part of my promotion and tenure file

Any other significant factors that influenced your decision to publish in F1000 Research? [Open text box]

**4) Once an F1000 Research article is published, it is only indexed on a bibliographic database (e.g. PubMed) once the article is approved following peer review. How important is it that your articles are indexed on a bibliographic database? [5-point scale for each, Very important, Important, Moderately Important, Slightly important, Not important]**

**If [rated very important or important], please provide any reasons why this is important (such as promotion and tenure) [Open text box].**

**5) The F1000 Research platform is a very transparent peer review system. The peer reviews are open as are reviewer’s names. How important is this transparency to you [5-point scale for each, Very important, Important, Moderately Important, Slightly important, Not important]**

**6) How likely would you submit future manuscripts to F1000 Research or another pre-print server? [5-point scale, Very likely, Likely, Neutral, Not likely, Very unlikely]**

**7) How likely would recommend F1000 research or another pre-print server to a colleague? [5-point scale, Very likely, Likely, Neutral, Not likely, Very unlikely]**

**8) Any further comments about your experiences (positive or negative) of submitting/publishing with F1000 Research? [Open text box]**
