# Supplementary material for: Who and why do researchers opt to publish in post-publication peer review platforms? - findings from a review and survey of F1000 Research
Source: F1000Res. 2018 Jun 27;7:920. [Version 1] doi: 10.12688/f1000research.15436.1 (PMC6053701; doi:10.12688/f1000research.15436.1)
Supplement: Peer review history

# Peer review: Referee response for version 1

**DOI:** 10.5256/f1000research.16821.r35534

**Published:** 2018-07-19

**Authors:** Anisa Rowhani-Farid

**Affiliations:** 1 Institute of Health and Biomedical Innovation (IHBI), Australian Centre for Health Services Innovation, Queensland University of Technology, Brisbane, Qld, Australia

Overall

 This is an excellent meta-research study of the open science platform, F1000 Research, using observational data and survey results.  The results of this study contribute to the shaping of journals’ conceptual framework, which influence their publishing practices, such as the adoption of post-publication peer-review, reducing publication costs, implementing open access policies, and reducing time between submission and publication.  I approve this study for publication, upon addressing the following points:

 Title

 The title was a bit confusing grammatically as the “who and why” would require differing conjugation and the “survey of F1000 Research” phrase might need to include “researchers”.  Here is a suggestion for the title:  A meta-research study of the post-publication platform, F1000 Research – who publishes there and why – findings from a review and survey. 

 Introduction

 This was good and covered the issue of “waste” in research because of the traditional journal publication system.  I wonder whether there is a dollar figure that demonstrates this waste?

 I think two other articles might be worthwhile citing in this introduction:

 Aleksic et al ^1^

 Tracz et al ^2^

 Methods and results

 The methods used are scientifically sound and clear.

 I think the ~one-month response period for the survey could be identified as a limitation of the study, especially given that there was only a 25-30% response rate, and after a few reminders too.  The emails could have ended up in researchers’ spam/clutter folders too.   

 I have had a look at the data shared.  The excel spread sheet contains the observational data of the articles published with F1000 Research from 13 July 2012 to 30 November 2017.  It might be worthwhile to export an anonymised copy of the survey data from REDCap to share as well.

 I am wondering why peer review was discontinued for those 36 articles.

 Discussion

 This was clear.  I have no further comments.

I have read this submission. I believe that I have an appropriate level of expertise to confirm that it is of an acceptable scientific standard.

## References

[1] AleksicJAlexaAAttwoodTKChue HongNDahlöMDaveyRDinkelHFörstnerKUGrigorovIHérichéJKLahtiLMacLeanDMarkieMLMolloyJSchneiderMVScottCSmith-UnnaRVieiraBMas part of the AllBio: Open Science & Reproducibility Best Practice Workshop: An open science peer review oath. *F1000Res*.2014;3: 10.12688/f1000research.5686.2 271 10.12688/f1000research.5686.2 25653839PMC4304228

[2] TraczVLawrenceR: Towards an open science publishing platform. *F1000Res*.2016;5: 10.12688/f1000research.7968.1 130 10.12688/f1000research.7968.1 26962436PMC4768651

# Peer review: Referee response for version 1

**DOI:** 10.5256/f1000research.16821.r35535

**Published:** 2018-07-10

**Authors:** David Mellor

**Affiliations:** 1 Center for Open Science, Charlottesville, VA, USA

The authors provide an appropriate and needed review of a leading platform in post publication peer review and preprints, F1000. They collect information on the country of origin, the "success" at being formally indexed, and survey authors on their experiences with the process (which is rarely shared about any other platform, so this information is particularly needed). My critiques below are all modest points for improvement. 

 "Traditionally, authors have not been able to add such research findings to their curriculum vitae and/or grant applications." The only harm implied by this lag is to the author. This roundabout system is also inefficient for reviewers, journals, and for dissemination to potentially interested readers. 

 "In some scientific fields such as pandemics or humanitarian emergencies, the time to deliver research findings may be as equally as important as research quality, and may be critical to health care provision." The authors should couch this as a balance between two conflicting needs: speed for potentially urgent information and ensuring that health-related information is of the highest possible quality. 

 "Open Science Initiative" I am not aware of a formal "initiative". Recommend revise to: "...new emerging options towards publication are being considered as part of the the "Open Science" movement."

 An additional benefit of Registered Reports is to address publication bias towards significant or novel results. 

 It might be worthwhile to mention that F1000 itself offers the RR workflow. https://blog.f1000.com/2017/10/12/transparency-meets-transparency/ 

 In the intro, the following citations would provide a bit more context for the reader 

1. Survey on open peer review: Attitudes and experience amongst editors, authors and reviewers ^1^
2.  Altmetric Scores, Citations, and Publication of Studies Posted as Prep ^2^
3. And for the section mentioning the rationale for Registered Reports, "Instead of “playing the game” it is time to change the rules: Registered Reports at AIMS Neuroscience and beyond" ^3^

"Non-responders were contacted periodically if a response to the survey was not received." Please provide your rule for re-contact (e.g. once per week until response was received or four contacts were made). 

 "All positive and negative experiences were independently reviewed by both authors and categorized into common topics. Any discrepancies were solved via discussion." What does this mean? That each free response, positive/negative opinion was scored by this study's authors (since you're describing a survey or authors, please clarify who the "authors" are in this sentence). 

 "with no more than 10% of the remaining articles published representing a different article type" Case report was slightly over 10% (this is obviously a nitpick, but it did lead me to wonder if I was looking at the correct column in the correct table). 

 "The majority of articles published received non-commercial funding" Recommend revise to "The majority of articles published reported receiving non-commercial funding..."

 "Economic status was classified according to the World Bank list of economies (June 2017)" Please provide a specific link to this list.

 "For the remaining 20% of articles, the lack of indexing was because peer review was incomplete (n=317), peer review had discontinued (n=36) or the article had been removed by authors (n=3)." Does "incomplete" include both articles that received poor reviews and articles that have received 1 or fewer reviews? Clarify the number of articles that received something equivalent to "rejections". I am casually familiar with F1000, perhaps more so than typical but less so than a frequent reader. It is unclear to me if "peer review ongoing" could ever revert to another status. Were there any articles that received multiple reviews that indicated poor article quality? Perhaps provide a bit more explanation of the F1000 workflow in the introduction and also define these categories a bit more precisely. 

 "29% (n=336), after one revision," There appears to be a math error here, probably should be 23%, but please check.

 Figure 2, "Recommendation" I think should be revised to "Recommendation by colleague", as all the other titles were self-explanatory (to me), but that one I had to open up the survey to understand what it meant (thanks for providing the survey!).

 "Authors found the process of nominating and reviewers agreeing to review challenging (n=9)" This sentence seems like you are inferring an opinion by 9 people to the entire author pool. I recommend leading the section "Experiences of those submitting articles to F1000 Research" with opinions that can be reliably noted by larger groups from within your sample, and then mention these small N opinions with appropriate caveats (e.g. "A few authors noted...."). Giving the Ns means you are not misleading anyone obviously, it just reads a bit odd to present this as a common experience. Likewise with the last sentence in that paragraph, "A number of authors [had various complains, n=5 and 2]." 

 "The speed and efficiency of publication (n=11) was the main reason that authors felt the experience was positive" change "main" to "most often noted in the free responses" (if that is what you mean by "main").

 "The peer review for about 15% of the articles had either been ongoing for more than a year or discontinued completely meaning that many of these articles may have contributed to the vast quantity of inaccessible unpublished literature (and the potential for publication bias) had these articles been subjected to the standard peer review before publication model." This sentence implies that the rest of the articles would have been published elsewhere if not submitted to F1000. I think that is overly optimistic. I would describe that 15% as a floor of that estimate. Also, this sentiment is likely to be met by a cynical reader (one who is more skeptical about the value of preprints than I am), that this is a good thing, that many published articles, and certainly many preprints, do not "deserve" to be published. I recommend addressing or acknowledging that sentiment here. 

 "There was also a general sense that the F1000 Research platform appeared to be ‘modern’ and yet was potentially ‘less attractive to the early career researcher’ because the need to publish in recognised journals with high impact factors is still considered the standard by the vast majority of researchers and institutes to gain promotion and tenure."

 ‘less attractive to the early career researcher’  Is that a quote from a survey respondent? State that in this part of the discussion, as that could also have come from other sources discussing the pressures on ECRs to publish in more "prestigious" venues.

I have read this submission. I believe that I have an appropriate level of expertise to confirm that it is of an acceptable scientific standard, however I have significant reservations, as outlined above.

## References

[1] Ross-HellauerTDeppeASchmidtB: Survey on open peer review: Attitudes and experience amongst editors, authors and reviewers. *PLoS One*.2017;12(12) : 10.1371/journal.pone.0189311 e0189311 10.1371/journal.pone.0189311 29236721PMC5728564

[2] SerghiouSIoannidisJ: Altmetric Scores, Citations, and Publication of Studies Posted as Preprints. *JAMA*.2018;319(4) : 10.1001/jama.2017.21168 10.1001/jama.2017.21168 PMC583356129362788

[3] D. ChambersCFeredoesED. MuthukumaraswamySJ. EtchellsP(1 Cardiff University Brain Research Imaging Centre, School of Psychology, Cardiff University): Instead of “playing the game” it is time to change the rules: Registered Reports at AIMS Neuroscience and beyond. *AIMS Neuroscience*.2014;1(1) : 10.3934/Neuroscience.2014.1.4 4-17 10.3934/Neuroscience.2014.1.4

# Peer review: Referee response for version 1

**DOI:** 10.5256/f1000research.16821.r35531

**Published:** 2018-07-04

**Authors:** Larry Peiperl

**Affiliations:** 1 Public Library of Science, San Francisco, CA, USA

F1000 research provides a platform for immediate publishing of research followed by open peer review and, if approved by reviewers, indexing in PubMed or other databases.  In the current article, the authors, who are leaders in the fields of metaresearch and journalology, provide a descriptive summary of the 1865 articles that were published in *F1000 Research* from earliest publication in July 2012 through November 2017, verifying peer review status through May 2018.  They also conduct an online survey in April-May 2018 through email requests to corresponding authors of articles that had been subject to external peer review.

 The results are descriptive and presented in summary format, which seems appropriate to the study design.  No statistical inference is undertaken.

 A spreadsheet of study-specified characteristics for all 1865 F1000 articles, and survey questions (not responses, which may possibly reflect an effort to preserve confidentiality of respondents) are provided as supplementary files.

 The bibliometric analysis appears straightforward. The authors may wish to clarify the following points, most of which pertain to the survey and interpretation of its (partially qualitative) results.

Methods

 1. “All positive and negative experiences were independently reviewed by both authors and categorized into common topics. Any discrepancies were solved via discussion.”  Can you provide more detail on how this qualitative analysis was performed? For example, were the “common topics” pre-defined?  If not, how did you arrive at the 3 thematic categories that you present in the Results?  Especially in light of the appropriately declared author competing interest, and the apparent relevance of the conclusions to the platform on which the authors are publishing the article, further information about how the study methodology may have supported objective interpretation of the qualitative data could strengthen the presentation.

Results

 2. Thirty-six articles had peer review “discontinued”. Under  what circumstances does discontinuation of peer review occur?

 3. Of 1476 unique corresponding author email addresses targeted in the survey, you received 296 responses, representing some 20% of the corresponding authors.  You identify the low response rate as a limitation of the study, but can you comment further on the representativeness of this sample?  Are you able to draw any useful conclusions based on the data you obtained for the full set of articles (year of publication, country income level, etc) regarding the extent to which responders may have differed from non-responders?  If such an analysis is not feasible, more caution would seem appropriate in drawing conclusions on the basis of the survey results.

Discussion

 The authors might clarify their reasoning at several points:

 4. Article: “There was also a sense that there was the potential for an article to become caught up in the process, immediate publication meant that there was limited scope to remove or submit elsewhere if peer reviewers could not be found or existing reviewers failed to provide subsequent reviews. While these criticisms may have reflected the experiences of some survey respondents, this process is not dissimilar from the many journals/publishers of standard journals which request names of peer reviewers, and in some instances release articles if peer reviewers cannot be found in a reasonable timeframe.”

 Comment: If an author believes that an article published prior to peer review in F1000 cannot then be submitted to another journal, that author’s situation would seem different from when a journal rejects an article after peer reviewers cannot be found.  In the latter situation, the author has the option to pursue indexing by submitting the article to another journal.  It’s not clear from this paragraph whether or not authors have this option for articles that haven’t passed peer review in F1000.

 5.  While there are some similarities, I’m not sure it’s fair to say that the PLOS partnership with bioRxiv “largely mimics the idea of the existing F1000 Research publication model.”   PLOS is partnering with bioRxiv in a way that other journals may choose to adapt, to complement the traditional journal publishing model by speeding dissemination while maintaining author choice regarding preprint posting and ultimate publication venue. Within this collaboration, bioRxiv does not function as an end-to-end publishing platform like F1000Research. Preprints posted to bioRxiv are not identified as having been submitted to a PLOS journal prior to publication in that journal, and authors are free to submit their posted work to another journal if the PLOS journal does not accept it.

 6. Article: “There still remains a level of dogma surrounding [preprint platform] use by many, and there remain concerns about the quality of the articles published on these platforms.”

 Comment:  It's not clear what you refer to as “dogma." The term seems dismissive, yet your research article identifies a number of reasonable concerns pertaining to preprint platform use.

I have read this submission. I believe that I have an appropriate level of expertise to confirm that it is of an acceptable scientific standard.
